# Phosphate Glass Microspheres with Silver Nanoparticles for Whispering Gallery Mode Resonators

**DOI:** 10.1021/acsanm.4c00652

**Published:** 2024-05-10

**Authors:** Foteini Dragosli, Ioannis Konidakis, Emmanuel Stratakis

**Affiliations:** Foundation for Research and Technology-Hellas (FORTH), Institute of Electronic Structure and Laser (IESL), 70013 Heraklion-Crete, Greece

**Keywords:** phosphate glass, microsphere resonators, silver nanoparticles, silver plasmon, post-glass melting treatment

## Abstract

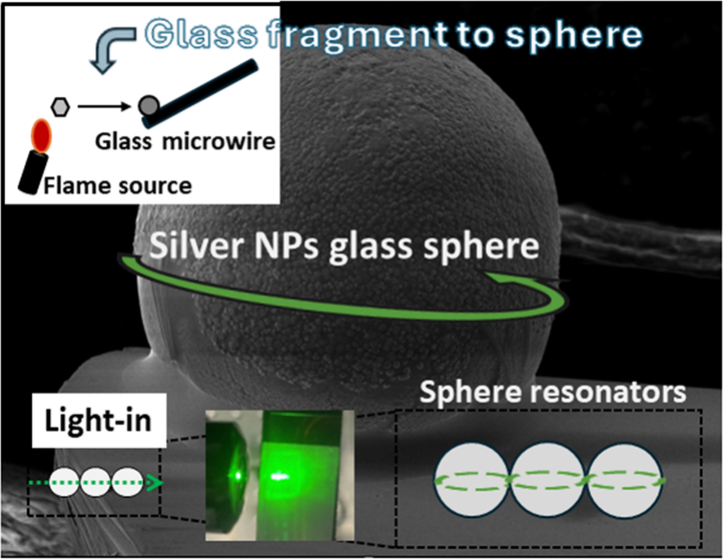

Glass microspheres have gained significant attention over the years in the field of photonics due to their application in whispering gallery mode (WGM) microresonator platforms. However, the synthesis of glass spheres in the micro regime remains challenging, while it relies mostly on complicated synthetic methods or sol–gel chemistry. Herein, we demonstrate the controlled formation of phosphate glass microspheres by means of a simple, fast, low-temperature, post-glass melting thermal treatment of previously quenched glass. Moreover, we report on the simultaneous formation of silver nanoparticles (AgNPs) on the surface of glass spheres upon the same treatment. The formation of metal nanoparticles onto the glass spheres induces attractive optical and plasmonic properties, believed to be suitable for WGM resonator-based applications, as well as a wide range of optoelectronic, photonic, and sensing applications.

## 1 Introduction

Glass microsphere resonators have been used in a wide variety of domains, from optical signal processing to lasing and biosensing.^1−7^ Because of their exceptional coupling efficiency and high Q values, glass microspheres are excellent candidates for use as narrow-linewidth optical filters, and as a result, their usage as distinctive tools in wavelength division multiplexing (WDM) applications has been addressed. As, for example, Yamaguchi et al. developed a whispering gallery mode (WGM) optical device with passive microspheres and nonlinear microspheres, allowing rapid refractive index modification with a controlled light signal.^1^ Moreover, in fiber loop lasers, a WGM resonator stabilizes narrow-linewidth lasing by opening the loops and inserting microspheres, tapered fibers, and angle-polished fibers, as described by Sprenger et al.^2^ On a rather different manner, fiber-coupled microspheres can be used for temperature measurements by observing the shift in WGM resonant wavelengths caused by circumference expansion. Along similar lines, a thermal point sensor was developed by Özel et al.,^3^ using a microsphere as an optical resonator and a hollow-core optical fiber as the detector. This setup allows heat detection with ±0.005 °C accuracy and 0.05 nm/°C sensitivity, covering 1000 °C. Other works describe the integration of microspheres for nanometric sensitive displacement platforms^4^ and photorefractive tuning of WGMs.^5^ Microspheres are also ideal for microphotonic biosensing due to their strong evanescent field, allowing interaction between internal WGMs and the environment. A waveguide-coupled microsphere-based biosensor was used by Keng and co-workers^6^ to identify a single virus in a SiO_2_ microsphere, allowing for the calculation of its volume and mass. Remarkably, sphere-based WGM architectures have been employed also for the development of lasing platforms with enhanced gain properties, opening new ways toward the realization of future tunable coherent light sources.^7^

Inorganic oxide glasses are commonly used materials for fabricating glass microsphere resonators due to their desirable optical and mechanical properties, such as low optical absorption, high transparency in the visible and near-infrared regions, and good thermal stability. Phosphate and silicate-based glasses are two examples of the most frequently used families. The phosphate glass is an example of a conventional oxide glass. It has numerous beneficial properties that make it suitable for use in glass resonators, including a long fluorescence lifetime, a large stimulated emission cross section, a large gain coefficient, moderate phonon energy, and low fluorescence quenching.^8^ Silicate is another example of a conventional oxide glass with characteristics including a small expansion coefficient, small dispersion, and outstanding chemical and thermal stability.^8^ Tellurite, on the other hand, is an example of a heavy metal oxide glass. It has good chemical stability, thermal stability, and mechanical properties and can also be fabricated by using a relatively simple process. Tellurite glass has a high refractive index and different structural units that is greatly reducing the quenching phenomenon.^8^

In terms of glass sphere fabrication procedures, there have been several proposed methods examined so far, but they all have significant disadvantages. For instance, protocols like the microwave plasma torch method or the sol–gel method only generate a small number of microspheres, and they both require expensive maintenance and complicated control procedures.^9−13^ Namely, in the first case, the experimental setup requires constant safety checks because high temperatures are required, and in the second case, several expensive chemicals are used, and it is crucial to monitor parameters like pH, temperature, and other variables.^9,10^ Moreover, methods like fine streaming into liquid nitrogen and the vertical tube furnace method also present the problems of limited material production, safety, and cost, with the additional problem that the microspheres produced by both of these methods have very specific characteristics in terms of their size and shape.^11,12^ Finally, the chemical foaming method, another method being used in the microsphere fabrication, in addition to all of the negative aspects already discussed, is also prone to environmental toxicity.^13^

Due to all of these drawbacks and challenges, a new quick, affordable, and effective method for creating microspheres is required to facilitate the production of glass microspheres for a variety of photonic applications. Based on this, in this work, we present the development of an advanced, fast, low-temperature, feasible post-melting thermal synthesis method for the development of silver phosphate (AgPO_3_) glass microspheres. The so-formed glass microspheres were subjected to scanning electron microscopy (SEM) analysis, which confirmed that the proposed technique allows us to control the size and shape of the microspheres. Moreover, it enables the formation of silver nanoparticles (AgNPs) and microclusters on microspheres surface. The latter were examined by optical absorbance and transmission electron microscopy (TEM) techniques, while their presence initiates interesting surface plasmon resonance effects and characteristic optical properties, targeting WGM resonators. Notably, it was revealed that without modifying the phosphate glass network, as confirmed by Raman spectroscopy, we observed that the AgNPs display optical and plasmonic properties, which are useful for a broad range of WGM applications.

## 2 Experimental Section

### 2.1 Synthesis of AgPO_3_ Glass and Microspheres

Silver metaphosphate glass, AgPO_3_, was prepared by melting equimolar amounts of high-purity AgPO_3_ (99.995%) and NH_4_H_2_PO_4_ (99.999%) dry powders, following a well-established synthesis procedure.^14^ In brief, following mixing, the melting batch was transferred to an electrical furnace initially held at 170 °C and gradually heated to ∼300 °C for the smooth removal of the volatile gas products. Then, the furnace temperature was raised to 350 °C and held steady to ensure melt homogeneity. AgPO_3_ glasses were obtained in the form of 1 mm thick disk specimens with a diameter of around 10 mm upon splat-quenching the melt between two silicon wafers. The employment of silicon wafers allows for the formation of smooth glass surfaces.

**Figure 1:**
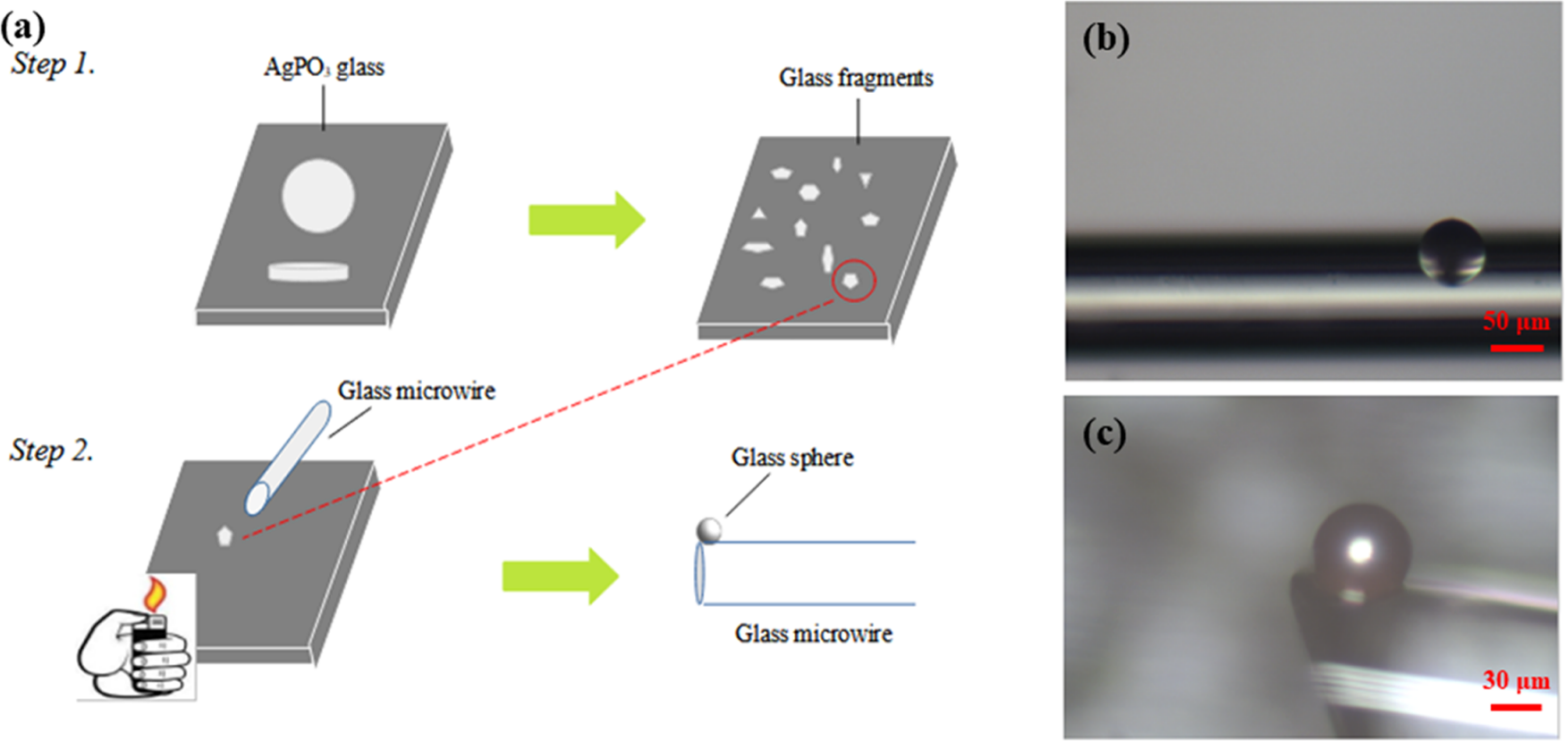
(a) Schematic representation of phosphate glass sphere synthesis started from the formation of AgPO_3_ glass fragments on a typical microscope slide (Step 1), followed by the heat treatment for the formation of the spheres (Step 2). Indicative optical microscopy photos of glass spheres with a diameter of 60 nm at lower (b) and higher (c) magnification.

For the formation of glass microspheres, following the typical splat-quenched glass preparation, the glass sample was shuttered to many little pieces, each one having an individual mass of a few micrograms (Step 1 in Figure 1a). Following this, in Step 2, one of the pieces is removed and placed on the surface of a silica wafer, as depicted schematically in Figure 1a. A standard lighter was employed as a heat source for the formation of the glass microsphere, namely, as the lighter approaches closely to the glass microfragment it transfers heat to it. When the lighter is positioned at around 5 mm from the glass microfragment and kept there for a few seconds, it raises the temperature of the glass near the glass transition temperature (192 °C).^14^ Consequently, the glass gains viscosity, while surface tension forces make the glass spherical. Meanwhile, a typical commercially available silica optical fiber is positioned a few millimeters away from the glass fragment (Figure 1a). During the melting of the glass fragment for the formation of the glass spheres, electrostatic forces enable the so-formed glass spheres to be attached close to the end face of the fiber, as shown in the optical microscopy photos in Figure 1b,c. Inspection of Figure 1b,c reveals the formation of well-shaped spherical phosphate glass spheres, which will be characterized further by means of scanning electron microscopy (SEM). Also, it is noted that the size of the developed spheres is directly proportional to the weight of the initial glass fragment, and thus, the proposed method provides a simple and direct tool for controlling the size of the spheres. Notably, the lighter heating few-seconds process must be repeated for every sphere that needs to be prepared, upon selecting the appropriate glass fragment.

### 2.2 Characterization

The samples were examined using scanning electron microscopes (JEOL, JSM-7000F and JSM-6490) equipped with an INCA PentaFET-x3 EDS detector for energy-dispersive X-ray spectroscopy. Moreover, the morphology of the so-formed AgNPs was studied by means of scanning transmission electron microscopy (STEM) operated in high-angle annular dark-field (HAADF) mode. Raman spectroscopy was used to study any potential changes to the glass network that might have occurred after the formation of AgNPs on the surfaces of the glass samples. Room-temperature Raman spectra with a resolution of 1 cm^–1^, through the utilization of a 532 nm laser line for excitation, were obtained at the backscattering geometry. The optical absorption characteristics of the glass samples with the presence of the AgNPs were examined using a PerkinElmer UV/vis (Lambda 950) spectrophotometer in the wavelength range of 280–850 nm.

## 3 Results and Discussion

**Figure 2:**
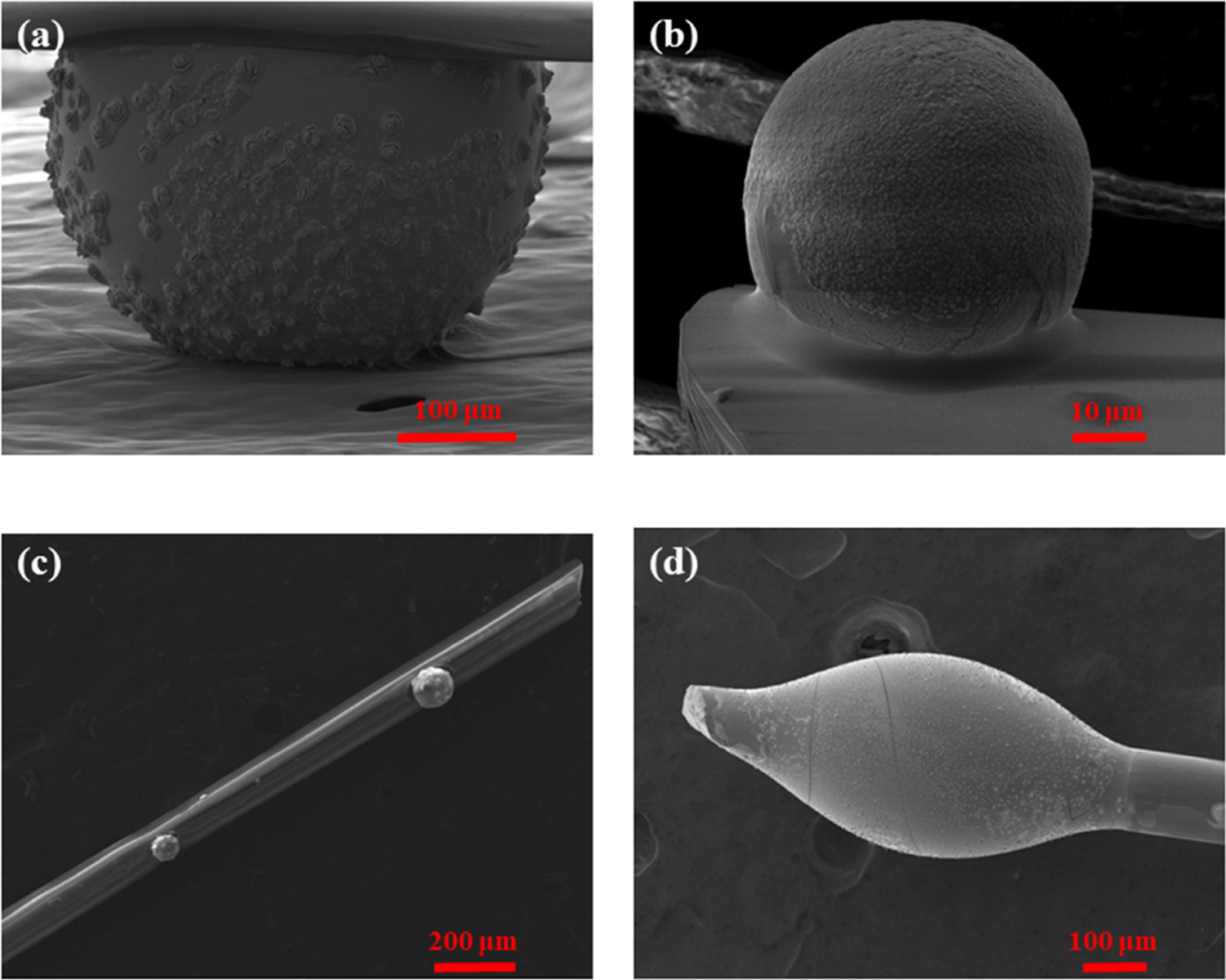
Indicative scanning electron microscopy (SEM) images of spheres with diameters of (a) ∼425 μm, (b) ∼50 μm, and (c) ∼80 μm and ∼60 μm. (d) SEM image of a sphere with different structure, i.e., oval with a vertical diameter of ∼300 μm.

Figure 2 presents indicative scanning electron microscopy (SEM) images of the developed AgPO_3_ spheres. In particular, Figure 2a–c depicts spheres of different diameters, whereas Figure 2d shows an oval-shaped sphere positioned around the optical fiber. Notably, depending on the weight of the employed glass fragment, the diameter of the fabricated sphere may vary from 10 up to 400 μm. The spheres can be easily detached from the holding silica fiber upon touching the contact point with a blade knife, so they can be employed for further characterization and integrated into photonic devices and platforms. For the oval-shaped example (Figure 2d), the horizontal diameter of the sphere is around 600 μm, which is directly related to the speed of pulling away the fiber from the heat source once the sphere is initially attached to the surface of the fiber. The vertical diameter of the depicted oval-shaped spheres is around 300 μm, i.e., depending mainly on the mass of the employed glass fragment. Moreover, inspection of SEM photos of Figure 2 reveals the presence of silver-based clusters and nanoparticles on the surface of the developed spheres.

**Figure 3:**
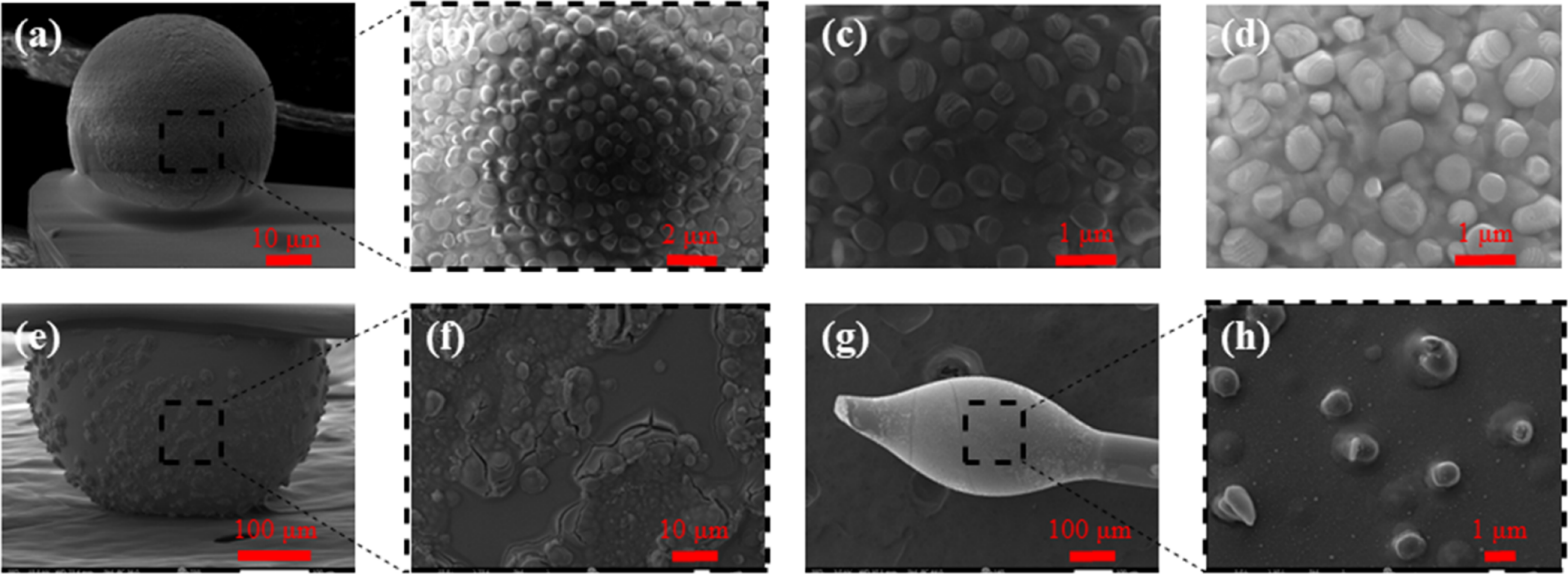
(a) SEM image of the 50 μm sphere and (b–d) the corresponding magnified SEM photos; (e) SEM image of the 425 sphere and (f) the corresponding magnified SEM image. (g) SEM image of the oval 300 μm sphere and (h) the corresponding magnified SEM image.

Figure 3 presents magnified SEM photos of the studied glass microspheres to elucidate on the nature of the AgNPs and clusters. Indeed, Figure 3a shows the medium-sized sphere of 50 μm, along with the corresponding magnified pictures of the surface (Figure 3b–d). The presence of randomly placed microclusters on the surface becomes apparent. Rather differently, Figure 3f demonstrates that on the surface of the larger glass sphere, instead of microclusters, the surface exhibits flake inhomogeneities and bursts. On the oval-shaped sphere, Figure 3h depicts the formation of microclusters, as was the case for the medium-sized spheres, but to a lesser extent as the obtained domains are less and distributed at longer distances. This observation is rationalized in terms of the forces from pulling away horizontally the optical fiber, while the glass is still soft and attached to the tip of the fiber. Overall, the obtained silver-based domains on the surface of the glass spheres emerge from the presence and agglomeration of AgNPs within the AgPO_3_ glass, existing prior to the post-melting thermal treatment for the formation of the spheres.

The presence of AgNPs within silver-based phosphate glasses has been demonstrated previously in some of our previous studies.^15,16^ In particular, following post-melting femtosecond (fs) laser treatment of the AgPO_3_ glass for the formation of periodic patterns on the surface, the formation of AgNPs was noticed due to the local heat transfer to the glass surface.^15^ Likewise, the presence of AgNPs was utilized for the formation of two-dimensional (2D) materials nano-heterojunctions upon incorporating few layers of MoS_2_ within the AgPO_3_ glass.^16^ In such configuration, the enhancement of the photoluminescence properties of the embedded 2D material was achieved due to the silver plasmon resonance of the AgNPs. Thus, in the present study, the employed thermal treatment causes agglomeration of the AgNPs for the formation of silver domains and clusters on the surface of the so-formed spheres. Nevertheless, in case that surface smoothening is required for performance optimization of WGM resonators, further annealing treatment near the glass transition temperature of the base glass^17^ would result in the encapsulation of the AgNPs beneath the glass surface, i.e., rendering the sphere surface smoother. To explore the nature of the obtained AgNPs, we performed optical spectroscopy and TEM studies on the employed glass.

**Figure 4:**
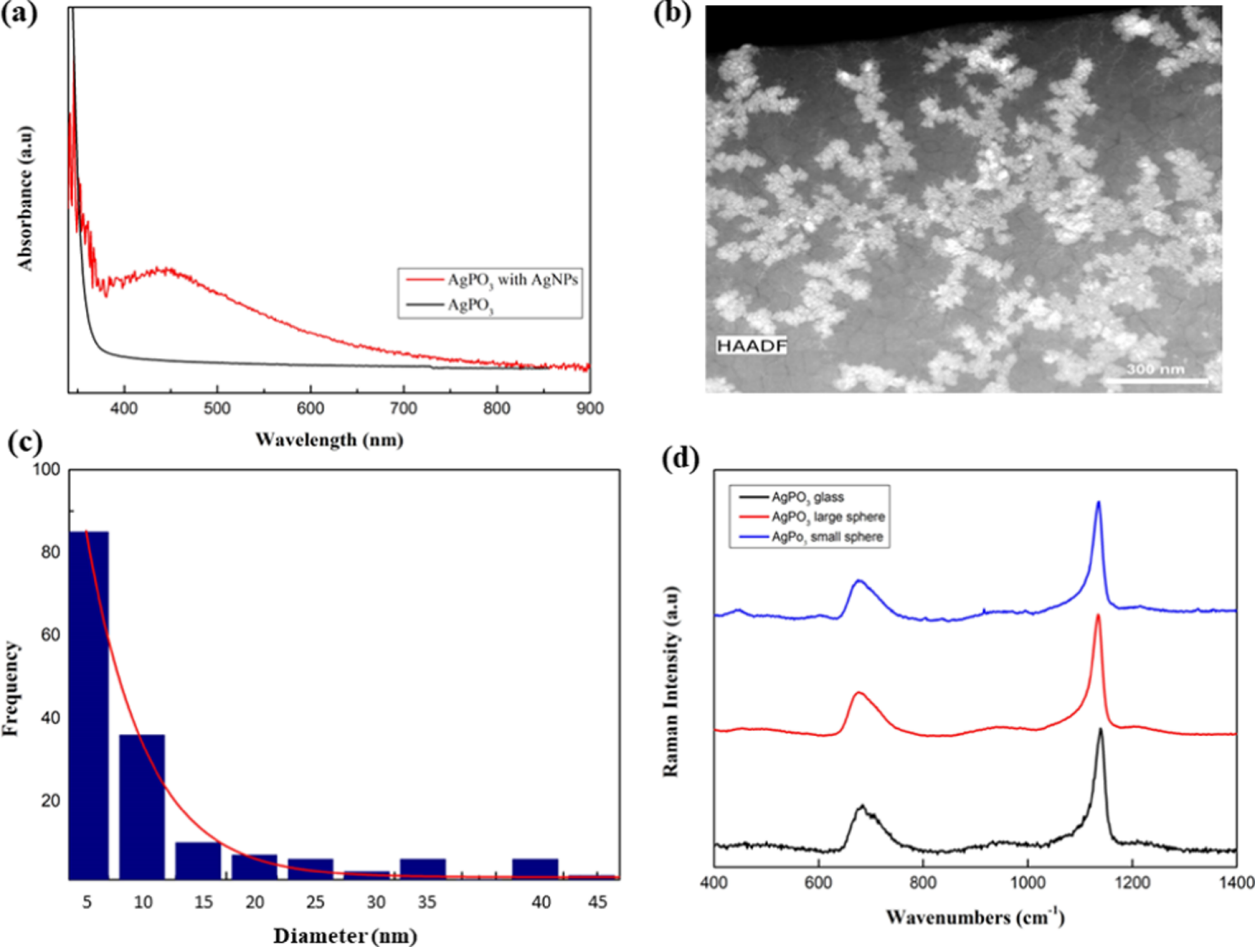
(a) Optical absorbance spectra of the glass microspheres with AgNPs (red line) and glass without AgNPs (black line). (b) Transmission electron microscopy (TEM) image of the AgNPs within the employed glass. (c) TEM analysis revealing AgNPs within the range 0–50 nm. (d) Room-temperature Raman spectra of the AgPO_3_ glass and two AgPO_3_ spheres with different sizes. For the sake of comparison, the Raman spectra are normalized on the ∼1140 cm^–1^ strongest band.

Figure 4a shows the optical absorbance of the AgPO_3_ glass employed in this study along with a reference absorbance profile of the same glass that was ultrafast quenched in order to prevent the formation of AgNPs. It is revealed that the former glass exhibits an absorbance peak at the 420 to 680 nm range, whereas such a peak is absent from the profile of the AgNPs-free glass. The obtained peak is attributed to the presence of AgNPs, while its broad profile implies a wide size distribution of the nanoparticles.^18^ This confirms that the glass used for the formation of the spheres contains AgNPs. Figure 4b presents a typical TEM image of the AgNPs, whereas the size distribution analysis of the particles is depicted in Figure 4c. Notably, most of the particles exhibit a diameter below 10 nm, whereas particles with diameters of up to 50 nm are present. As discussed previously, the presence of the AgNPs within the phosphate glass results in the formation of the obtained silver microcluster domains on the surfaces of the developed spheres (Figure 3a–d). Interestingly enough, the formation of such domains appears to weaken when the size of the sphere increases. This is rationalized in terms of the reduction of the electrostatic forces applied to the nanoparticles once the distances from each other increase. Consequently, when the sphere diameter exceeds 400 μm, the agglomeration of AgNPs diminishes as revealed from the SEM photos (Figure 3e,f). In that case, only surface inhomogeneities are observed, caused by the surface tension of the glass during the post-melting heat treatment for the formation of the spheres. Finally, in the medium-sized oval-shaped spheres (Figure 3g,h), only partial formation of silver clusters is noticed.

We move on now to consider any potential alterations in the phosphate glass network upon the transition from glass fragment to sphere and the formation of silver microclusters on the surface. Figure 4d presents room-temperature Raman spectra of the AgPO_3_ glass, along with the corresponding spectra of a large sphere (exceeding 400 μm) and a small sphere of 50 μm. Phosphate glass network consists mainly of phosphate chains formed by linked tetrahedral units with bridging and nonbridging (terminal) oxygen atoms.^17,19^ In particular, the strongest band, at around ∼1140 cm^–1^, originates from the symmetric stretching vibration of terminal PO_2_^–^ groups, or v_s_(PO_2_^–^), while the broader band, at around ∼680 cm^–1^, is caused by the stretching of P–O–P bridges inside the phosphate backbone, or v_s_(P–O–P). The relative intensities of these two bands depict an immediate signature of the alteration on the population of bridging and terminal entities. Examination of Figure 4d demonstrates similar relative intensities of the two main bands, implying that despite the transformation to glass spheres and the agglomeration of AgNPs, the phosphate network maintains its structural characteristics.

**Figure 5:**
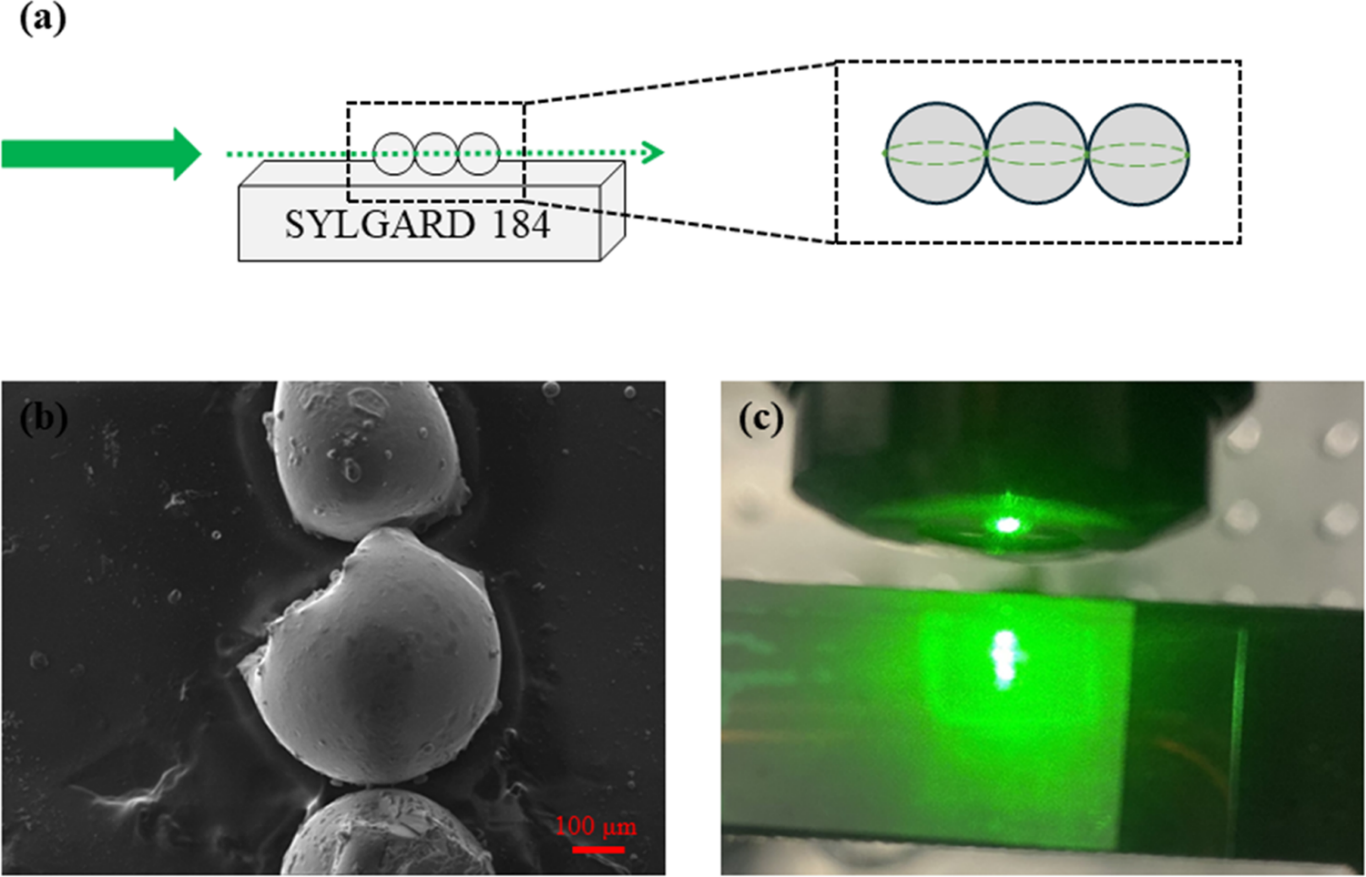
(a) Architecture of the microsphere-based waveguide along with potential orientation of WGM resonances. (b) SEM image of the three microspheres placed on the elastomer, i.e., partially immersed. (c) Light scattering occurred within the microsphere-based waveguide upon CW green laser excitation (512 nm).

We conclude our work by demonstrating the light scattering effects occurring within the fabricated microspheres. Particularly, as schematically indicated in Figure 5a, we have placed a few spheres on a silica elastomer. We used SYLGARD 184 as the host elastomer. Figure 5b depicts the corresponding SEM photograph of the architecture. Moreover, Figure 5c presents the spheres under CW green laser irradiation at 512 nm. The laser beam is focused on the first microsphere through a microscope objective lens. It becomes apparent that all three positioned spheres exhibit bright green light luminescence upon absorbing light from the laser source. Moreover, the presence of silver NPs both within and on the surface of the microspheres is believed to enhance light scattering in the green light region due to surface plasmon resonance effects.^20^ Notably, similar plasmon resonance features could be induced upon the introduction of another metal within the glass, as, for instance, gold. Figure 5a also schematically depicts the presence and potential orientation of WGM resonances within the constructed combined spheres architecture. We believe that such device architecture, and similar ones, could pave the way toward the development of advanced light scattering photonic platforms and WGM applications, including lasing platforms by means of introducing active materials like erbium and ytterbium within the binary phosphate glass.

## 4 Conclusions

In conclusion, we presented the development of a fast, simple, and low-temperature approach for the synthesis of glass microspheres. The described approach enables the synthesis of glass microspheres with controlled size and shape upon changing the parameters during the postmelting formation process. In addition, it was shown that the presence of AgNPs within the employed silver phosphate glass resulted in the formation of silver-rich domains on the surface of the so-formed spheres. The obtained cluster formation was caused during the post-melting thermal treatment of the glass for achieving the spherical shape and due to the agglomeration of the pre-existing AgNPs within the base glass. The size and population of these silver-rich domains were found to be related to the size and shape of the developed sphere. Notably, it was shown that the glass retained its structural characteristics throughout the transformation to glass spheres despite the presence of spatially distributed clusters on the surface of the spheres. Finally, a brief example of the light scattering properties of the silver-rich glass microspheres was demonstrated. The findings of the present study pave the way toward novel synthesis fabrication protocols of glass microspheres with AgNPs targeting advanced WGM resonator platforms, while it remains a continuous challenge to further optimize performance and photonic device architectures.

## References

[ref1] Yamaguchi K.; Fujii M.; Haraguchi M.; Okamoto T.; Fukui M. Nonlinear trimer resonators for compact ultra-fast switching. Opt. Express 2009, 17, 23204 10.1364/OE.17.023204. 20052247

[ref2] Sprenger B.; Schwefel H. G.; Wang L. J. Whispering-gallery-mode-resonator-stabilized narrow-linewidth fiber loop laser. Opt. Lett. 2009, 34, 3370 10.1364/OL.34.003370. 19881597

[ref3] Özel B.; Nett R.; Weigel T.; Schweiger G.; Ostendorf A. Temperature sensing by using whispering gallery modes with hollow core fibers. Meas. Sci. Technol. 2010, 21, 094015 10.1088/0957-0233/21/9/094015.

[ref4] Kosma K.; Konidakis I.; Pissadakis S. Photorefractive tuning of whispering gallery modes of a spherical resonator integrated inside a microstructured optical fibre. Eur. Phys. J. Spec. Top. 2014, 223, 2035 10.1140/epjst/e2014-02246-3.

[ref5] Kosma K.; Schuster K.; Kobelke J.; Pissadakis S. An “in-fiber” whispering-gallery-mode bi-sphere resonator, sensitive to nanometric displacements. Appl. Phys. B 2018, 124, 1 10.1007/s00340-017-6866-9.

[ref6] Vollmer F.; Arnold S.; Keng D. Single virus detection from the reactive shift of a whispering-gallery mode. Proc. Natl. Acad. Sci. U.S.A. 2008, 105, 20701 10.1073/pnas.0808988106. 19075225 PMC2603258

[ref7] Duan R.; Zhang Z.; Xiao L.; Zhao X.; Thung Y. T.; Ding L.; Liu Z.; Yang J.; Ta V. D.; Sun H. Ultralow-threshold and high-quality whispering-gallery-mode lasing from colloidal core/hybrid-shell quantum wells. Adv. Mater. 2022, 34, 2108884 10.1002/adma.202108884. 34997633

[ref8] Yu J.; Lewis E.; Farrell G.; Wang P. Compound glass microsphere resonator devices. Micromachines 2018, 9, 356 10.3390/mi9070356. 30424289 PMC6082264

[ref9] Shen Q.; Huang R.; Xu Z.; Hua W. Numerical 3D modeling: microwave plasma torch at intermediate pressure. Appl. Sci. 2020, 10, 5393 10.3390/app10155393.

[ref10] Hossain K. M. Z.; Patel U.; Ahmed I. Development of microspheres for biomedical applications: a review. Prog. Biomater. 2015, 4, 1 10.1007/s40204-014-0033-8. 29470791 PMC5151111

[ref11] Liu G.; Miao X.; Fan W.; Crawford R.; Xiao Y. Porous PLGA microspheres effectively loaded with BSA protein by electrospraying combined with phase separation in liquid nitrogen. J. Biomimetic Biomater. Biomed. Eng. 2010, 6, 1 10.4028/www.scientific.net/JBBTE.6.1.

[ref12] Ward J. M.; Wu Y.; Khalfi K.; Chormaic S. N. Short vertical tube furnace for the fabrication of doped glass microsphere lasers. Rev. Sci. Instrum. 2010, 81, 073106 10.1063/1.3455198. 20687704

[ref13] Xie J.; Chen L.; Xie H.; Zhou J.; Liu G. The application of chemical foaming method in the fabrication of micro glass hemisphere resonator. Micromachines 2018, 9, 42 10.3390/mi9020042. 30393318 PMC6187607

[ref14] Konidakis I.; Brintakis K.; Kostopoulou A.; Demeridou I.; Kavatzikidou P.; Stratakis E. Highly luminescent and ultrastable cesium lead bromide perovskite patterns generated in phosphate glass matrices. Nanoscale 2020, 12, 13697 10.1039/D0NR03254A. 32573581

[ref15] Konidakis I.; Skoulas E.; Papadopoulos A.; Serpetzoglou E.; Margariti E.; Stratakis E. Erasable and rewritable laser-induced gratings on silver phosphate glass. Appl. Phys. A 2018, 124, 1 10.1007/s00339-018-2267-0.

[ref16] Sarkar A. S.; Konidakis I.; Demeridou I.; Serpetzoglou E.; Kioseoglou G.; Stratakis E. Robust B-exciton emission at room temperature in few-layers of MoS_2_:Ag nanoheterojunctions embedded into a glass matrix. Sci. Rep. 2020, 10, 15697 10.1038/s41598-020-72899-3. 32973224 PMC7518262

[ref17] Milenko K.; Konidakis I.; Pissadakis S. Silver iodide phosphate glass microsphere resonator integrated on an optical fiber taper. Opt. Lett. 2016, 41, 2185 10.1364/OL.41.002185. 27176958

[ref18] Zong R.; Wang X.; Shi S.; Zhu Y. Kinetically controlled seed-mediated growth of narrow dispersed silver nanoparticles up to 120 nm: secondary nucleation, size focusing, and Ostwald ripening. Phys. Chem. Chem. Phys. 2014, 16, 4236 10.1039/c3cp54846e. 24452515

[ref19] Konidakis I.; Karagiannaki A.; Stratakis E. Advanced composite glasses with metallic, perovskite, and two-dimensional nanocrystals for optoelectronic and photonic applications. Nanoscale 2022, 14, 2966 10.1039/D1NR07711B. 35142770

[ref20] Huang C.-L.; Huang H. J.; Chen S.-H.; Huang Y.-S.; Kao P.-C.; Chau Y. -F. C.; Chiang H.-P. Localized surface plasmon resonance enhanced by the light-scattering property of silver nanoparticles for improved luminescence of polymer light-emitting diodes. J. Ind. Eng. Chem. 2021, 103, 283 10.1016/j.jiec.2021.07.044.

